# A novel prediction method for intracerebral hemorrhage-associated pneumonia: A single center analysis

**DOI:** 10.1371/journal.pone.0318455

**Published:** 2025-02-20

**Authors:** Ya-ming Li, Yue Chen, Mei-fen Yao, Guo-jiang Wang, Yi-ni Pan, Hui Chen, Jian-hua Xu

**Affiliations:** Department of Neurology, Jiading District Central Hospital Affiliated Shanghai University of Medicine & Health Sciences, Shanghai, China; UCSF: University of California San Francisco, UNITED STATES OF AMERICA

## Abstract

Stroke-associated pneumonia (SAP) is a common complication leading to death and disability after a stroke. Currently, more studies tend to focus on stroke-associated pneumonia in patients with ischemic stroke, while there are few studies on predictors of intracerebral hemorrhage-associated pneumonia (ICHAP). It is necessary to discover new predictors to build more accurate prediction models for ICHAP. We continuously collected 498 patients with acute intracerebral hemorrhage and then divided them into ICHAP and non-ICHAP groups. Then we conducted univariate analyses and multivariate regression analyses on the collected data. Afterward, the new predictors of ICHAP were found and the predictive model was designed. Of the 498 patients, 158 were diagnosed with ICHAP. Advanced age (odds ratio =  1.031; 95% confidence interval, 1.015–1.047; P <  0.001), high NIHSS score (odds ratio =  1.081; 95% confidence interval, 1.038–1.126; P <  0.001), dysphagia (odds ratio =  4.191; 95% confidence interval, 2.240–7.841 P <  0.001), and fast pulse (odds ratio =  1.038; 95% confidence interval, 1.019–1.057; P <  0.001) were risk factors for ICHAP. The predictive model (P <  0.001) included age, NIHSS, dysphagia, and pulse. After that, the receiver operating characteristic (ROC) curve and the corresponding area under the curve (AUC) were used to measure their predictive accuracy. The prediction ability of the model (AUC: 0.819) which contained age, NIHSS, dysphagia, and pulse was higher than that of advanced age (AUC = 0.670), high NIHSS score (AUC = 0.761), and fast pulse (AUC = 0.609). The predictive accuracy of the model was 81.5%. These findings might help identify high-risk patients for ICHAP and provide a reference for the timely preventive use of antibiotics.

## Introduction

Stroke-associated pneumonia (SAP) refers to the spectrum of pneumonia complicating the first 7 days after stroke onset. The incidence of SAP is 2.4% to 47%, which is a common complication after a stroke [1–3]. SAP can worsen the outcome of stroke and increase the incidence of severe disability. Even, some patients with severe stroke may die from the cause of the pneumonia [4,5]. However, most of the above studies were on stroke-associated pneumonia in patients with ischemic stroke. There are relatively few studies on intracerebral hemorrhage-associated pneumonia (ICHAP). Factors identified that may be used to predict intracerebral hemorrhage-associated pneumonia include older age, National Institutes of Health Stroke Scale (NIHSS), dysphagia, patients with coma, pre-stroke modified Rankin Scale (mRS), C-reactive protein levels, serum urea, cognitive impairment, etc [6–11]. However, a method capable of accurately predicting ICHAP has not been established. Moreover, for patients with acute intracerebral hemorrhage, routine examinations at admission have many more indicators than these predictive indicators. Therefore, it is necessary to discover new predictors in the routine examinations of these patients, and then combine the new factors with known predictors to form an accurate ICHAP prediction method.

In this study, we intended to collect and analyze the indicators included in routine admission examinations to identify rare and undiscovered factors that might be associated with ICHAP. Then, we would use these new factors to design a simple and practical method for predicting ICHAP.

## Methods

### Patients and population

Patients with acute intracerebral hemorrhage admitted to the department of neurology of Jiading District Central Hospital Affiliated Shanghai University of Medicine & Health Sciences from December 18, 2015, to December 18, 2020, were continuously collected. Patients were included in the study if they (1) had an acute onset of intracerebral hemorrhage confirmed by neuroimaging, (2) were not infected within 2 weeks before admission, (3) were hospitalized within 48 hours after the onset of the intracerebral hemorrhage symptoms, and (4) gave informed consent. Patients were excluded from the study if they (1) were using antibiotics, (2) had severe liver dysfunctions (3) had serious hematologic diseases. The data was accessed on January 17, 2022.

The ethical approval of this study was approved by the local ethics committee of Jiading District Central Hospital Affiliated Shanghai University of Medicine & Health Sciences (NO. 2021K03), Shanghai, China. In addition, the study obtained the written informed consent of all involved patients or their legal representatives.

### Clinical management and data

At the time of admission, all patients were determined whether they had pre-stroke infections through whether the patients had respiratory infection symptoms (e.g., cough, sputum, fever, etc.) before the onset of stroke symptoms and the blood test reports (including blood routine examination, C-reactive protein (CRP), etc.) of the emergency department. For a patient without pre-stroke infections, a neurologist was responsible for detailed recording of the patient’s demographic and clinical data, including gender, age, pulse at admission, blood pressure at admission, time of onset of intracerebral hemorrhage symptoms, smoking status, alcoholism, drug use, and disease history (e.g., hypertension, diabetes mellitus, atrial fibrillation or cardiac valve disease, chronic obstructive pulmonary disease, cerebral infarction, dementia, intracerebral hemorrhage, myocardial infarction, etc.). If the patient had a history of dementia, the neurologist evaluated the patient. The diagnosis of dementia was made by combining the patient’s clinical manifestations and auxiliary examination results. For example, a patient had at least two neurological impairments in memory, language, visual-spatial skills, executive function, application, and calculation, which seriously affected the patient’s daily or social ability to be considered as possible dementia. And, the relevant scales (Mini-mental State Examination (MMSE) [12] <  27 points; Montreal Cognitive Assessment (MoCA) [13] <  26 points) and brain nuclear magnetic resonance (MRI) scan results (temporal lobe and hippocampus atrophy, or frontal lobe and/or anterior temporal lobe atrophy) were used for comprehensive diagnosis. Next, the physician performed stroke-related assessments. The severity of the stroke, level of consciousness, and dysphagia were assessed by the neurologist using the National Institute of Health Stroke Scale (NIHSS) [14], Glasgow Coma Scale (GCS) [15], and Water Swallow Test (WST) [16], respectively. And, a patient with a WST evaluation result greater than 2 was considered to have dysphagia. Moreover, the modified Rankin Scale (mRS) [17] was used to evaluate the state of neurological function before the onset of the stroke. In addition, venous blood was taken from all patients at admission for routine laboratory measurements, including serum uric acid, serum urea, serum creatinine, blood glucose, low-density lipoprotein, international normalized ratio (INR), and so on. Each patient was examined and treated under the intracerebral hemorrhage treatment guidelines [18] by professional and trained neurologists during hospitalization. Subsequently, the body temperature, respiratory symptoms, physical examination, and auxiliary examination results of each hospitalized patient were observed and recorded within 7 days after the onset of stroke symptoms.

### Outcome measures

The spectrum of lower respiratory tract infections within the first 7 days after the onset of intracerebral hemorrhage was considered to be ICHAP [19]. Furthermore, the spectrum was defined as leukocytosis (>12000 × 10^9/L cells) or leucopenia (<4000 × 10^9/L cells) and/or fever (>38°C), and at least two of the following: (1) New purulent sputum, changes in sputum characteristics, or increased respiratory secretions, or increased demand for sputum suction; (2) New onset or worsening cough, or shortness of breath, or dyspnea; (3) Bronchial breath sounds, or rales; (4) Worsening gas exchange, increased oxygen demands. Moreover, when additionally typical chest X-ray or computed tomography (CT) changes were present [19,20], ICHAP was diagnosed.

### Statistical analysis

SPSS (Statistical Product and Service Solutions) software package version 22.0 was used to collect and analyze the data. If the continuous variables conformed to the normal distributions, the variables were expressed as means ±  standard deviations. On the contrary, if the continuous variables did not conform to the normal distributions, the variables were expressed as median ±  interquartile range. According to the nature of the variables, Pearson’s chi-squared tests or Mann-Whitney U tests were used for univariate analyses. The factors (P < 0.10) in the univariate analyses were included in multivariate logistic regression analysis. The collinearity diagnosis of these factors was made. The multivariate logistic regression analysis (using the ‘ Forward: LR ‘ method) was used to achieve risk factors and establish the prediction model. The model was detected by the likelihood ratio test. The predictive abilities of different indicators and the ICHAP model were measured by the receiver operating characteristic (ROC) curve and the area under the curve (AUC). The Alpha-error level was set at P = 0.05.

## Results

**Fig 1:**
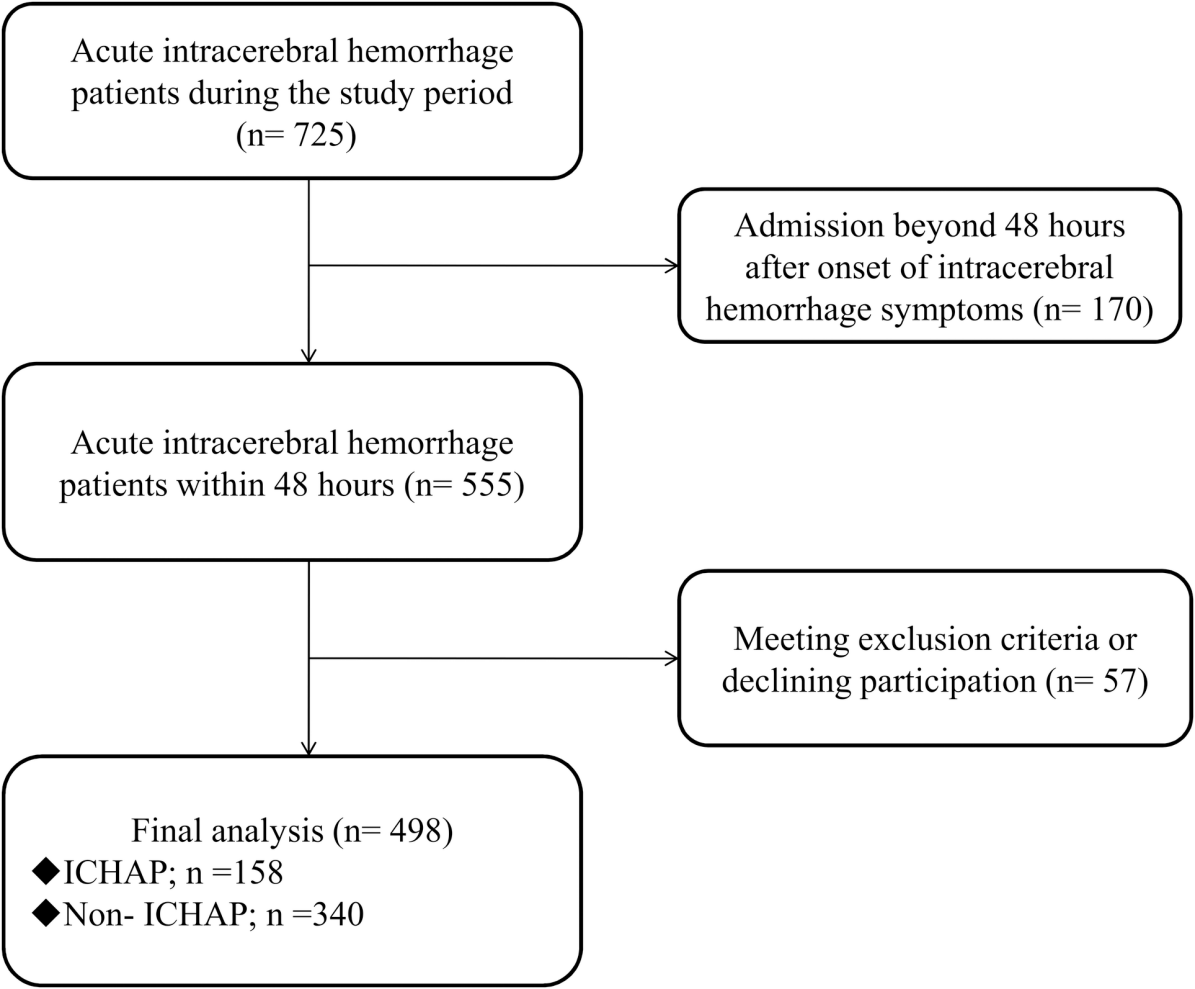
The flow of patients. ICHAP, intracerebral hemorrhage-associated pneumonia.

During the study period, a total of 725 patients were hospitalized for acute intracerebral hemorrhage. After screening, 498 patients (68.69%) met the inclusion criteria and agreed to participate in this study. These patients included 327 males, accounting for 65.66%, and 171 females, accounting for 34.34%. The youngest of the subjects was 19 years old, the oldest was 97 years old, and the average age was 66 years old (Fig 1).

The baseline characteristics between the ICHAP group and the non-ICHAP group were shown in Table 1. There were 158 patients with ICHAP within 7 days of the onset of stroke, with an incidence of 31.73%. The ICHAP group presented with older age and higher rates of dysphagia, intracerebral hemorrhage, and atrial fibrillation or cardiac valve disease history. And, admission NIHSS, admission pulse, INR, blood glucose, serum creatinine, serum urea, and serum uric acid levels were also higher in the ICHAP group than in the non-ICHAP group. Moreover, admission GCS and levels of low-density lipoprotein in the infected group were lower than those in the non-infected group. In addition, there was also a statistically significant difference in the pre-stroke mRS score between the infected and non-infected groups.

**Table 1:** Baseline characteristics of patients with ICHAP and non-ICHAP.

	Non-ICHAP (n = 1907)	ICHAP (n = 459)	*P* Value
Sex, male (%)	220(64.7)	107(67.7)	0.510
Age, y [IQR]	63 [52–74]	77 [60–85]	<0.001
History			
Hypertension (%)	243(71.5)	109(69.0)	0.571
Diabetes mellitus (%)	49(14.4)	23(14.6)	0.966
Atrial fibrillation or Cardiac valve disease (%)	2(0.6)	7(4.4)	<0.05
COPD (%)	2(0.6)	5(3.2)	0.062
Cerebral infarction (%)	46(13.5)	30(19.0)	0.115
Alcoholism (%)	63(18.5)	30(19.0)	0.903
Current smoker (%)	79(23.2)	31(19.6)	0.365
Dementia (%)	2(0.6)	5(3.2)	0.062
Intracerebral hemorrhage (%)	20(5.9)	19(12.0)	<0.05
Myocardial infarction (%)	3(0.9)	1(0.6)	1.000
Assessment at admission			
NIHSS score [IQR]	3.0[1.0–8.0]	11.0 [5.0–19.0]	<0.001
GCS [IQR]	15.0[14.0–15.0]	12.5 [8.0–15.0]	<0.001
Dysphagia (%)	37(10.9)	90(57.0)	<0.001
Admission systolic blood pressure (mmHg)	156 ± 24	159 ± 23	0.170
Admission Diastolic Blood Pressure (mmHg) [IQR]	86 [78 -96]	86 [77–95]	0.584
Admission pulse (times/min)	76 [68–82]	80 [72–88]	<0.001
Pre-stroke mRS score [IQR]	0.0 [0.0–1.0]	0.0 [0.0–1.0]	<0.05
Laboratory indicators			
INR [IQR]	0.92 [0.88–0.97]	0.96 [0.91–1.02]	<0.001
Blood glucose (mmol/L) [IQR]	5.70 [5.05–6.80]	6.33 [5.60–7.60]	<0.001
Low-density lipoprotein (mmol/L) [IQR]	2.80 [2.29–3.51]	2.59 [2.01–3.34]	<0.05
Serum creatinine (umol/L) [IQR]	71.00 [59.00–85.00]	78.00 [63.48–100.01]	<0.001
Serum urea (mmol/L) [IQR]	5.00 [4.10–6.20]	5.85 [4.60–7.83]	<0.001
Serum uric acid (umol/L)	312.71 ± 101.26	341.52 ± 138.65	<0.05

*IQR, interquartile range; COPD, chronic obstructive pulmonary disease; NIHSS, National Institutes of Health Stroke Scale; GCS, Glasgow Coma Scale; mRS, modified Rankin Scale; INR, international normalized ratio;*

Then, the indicators (p <  0.10) in the univariate analysis of the ICHAP group and the non-ICHAP group were included in the logistic regression model (using the ‘ Forward: LR ‘ method) for logistic regression. The result showed that age (odds ratio =  1.031; 95% confidence interval, 1.015–1.047; P <  0.001), NIHSS (odds ratio =  1.081; 95% confidence interval, 1.038–1.126; P <  0.001), dysphagia (odds ratio =  4.191; 95% confidence interval, 2.240–7.841 P <  0.001), and pulse (odds ratio =  1.038; 95% confidence interval, 1.019–1.057; P <  0.001) were risk factors for ICHAP. And, the variables that entered the model were age, NIHSS, dysphagia, and pulse (Table 2).

**Table 2:** Logistic regression model results.

	B	*P* Value	OR
Age	0.031	0.000	1.031
NIHSS	0.078	0.000	1.081
Dysphagia	1.433	0.000	4.191
Pulse	0.037	0.000	1.038
Constant	-6.831	0.000	0.001

*NIHSS, National institutes of health stroke scale; B, regression coefficient; OR, odds ratio.*

After that, all variables entering the model were assigned (Table 3). The regression model was: Logit (P) =  -6.831 + 0.031X_1_ + 0.078X_2_ + 1.433X_3_ + 0.037X_4_. And, we conducted a likelihood ratio test on the model, and the result showed that the model was significant (P <  0.001). Moreover, the prediction results of the model for the occurrence of ICHAP (a P >  0.50 was deemed to be ICHAP) showed that 94 of 158 SAP patients were correctly predicted; among 340 non-SAP patients, 312 were correctly predicted. The prediction accuracy of the model can reach 81.5%.

**Table 3:** Variable assignments.

	Variable	Remarks
Age	X_1_	–
NIHSS	X_2_	–
Dysphagia	X_3_	0 = “NO”, 1 = “Yes”
Pulse	X_4_	–

*NIHSS, National Institutes of Health Stroke Scale;*

**Fig 2:**
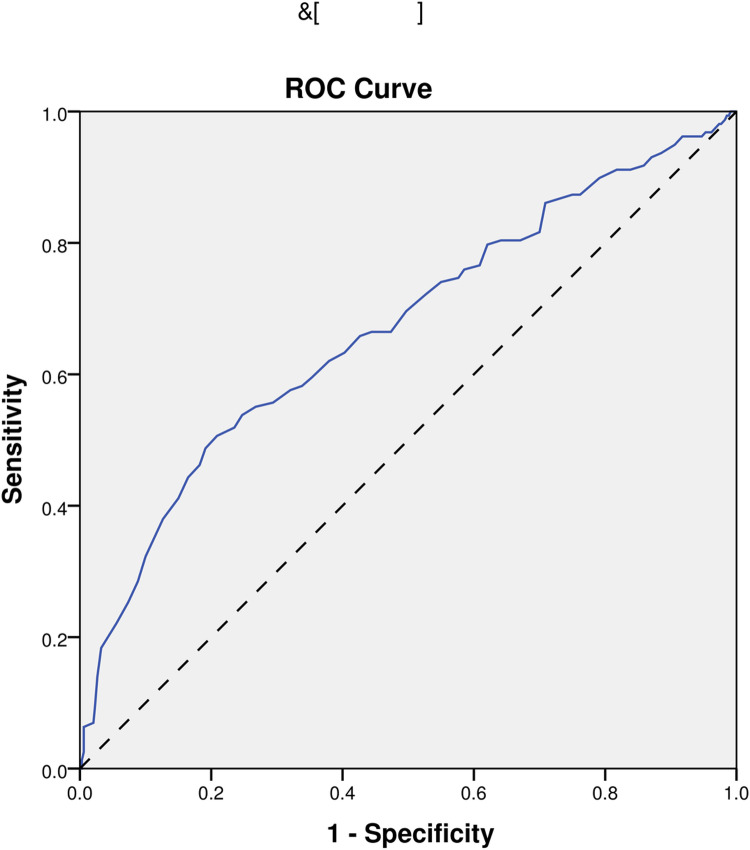
ROC curve of age. ROC, receiver-operating characteristic.

**Fig 3:**
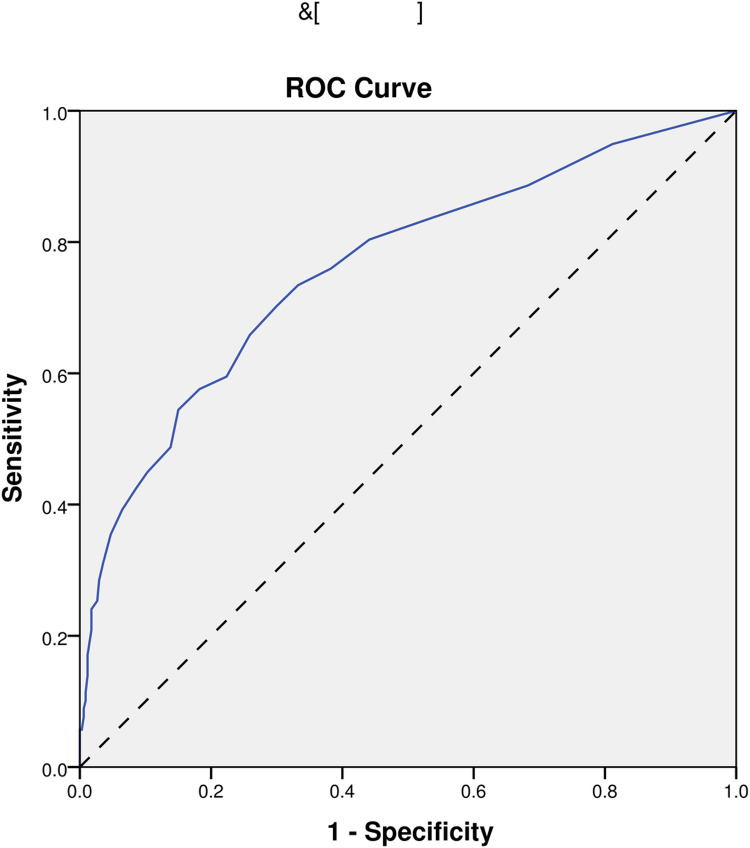
ROC curve of NIHSS. ROC, receiver-operating characteristic.

**Fig 4:**
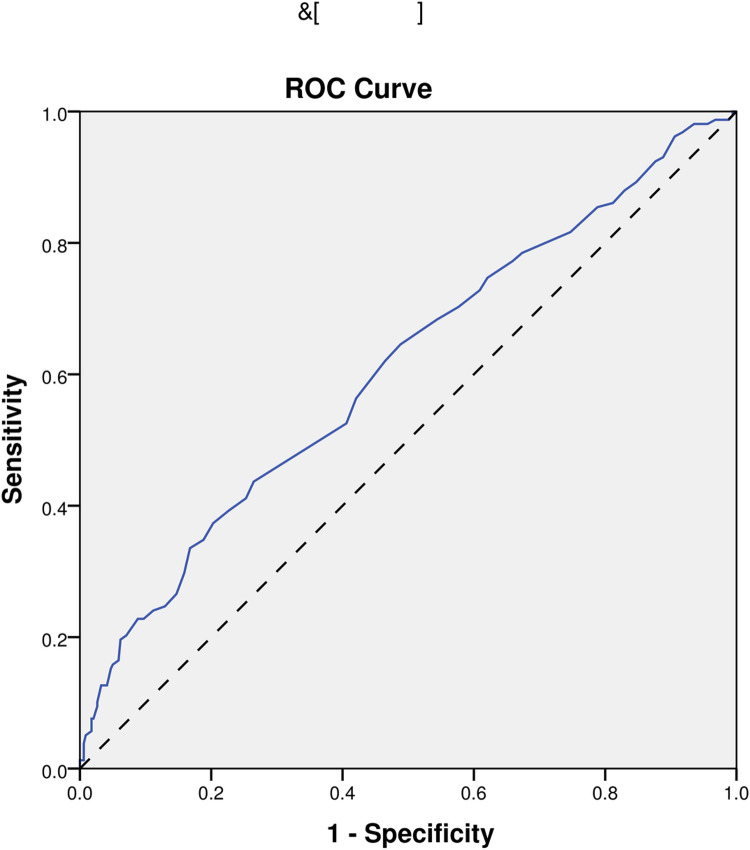
ROC curve of pulse. ROC, receiver-operating characteristic.

**Fig 5:**
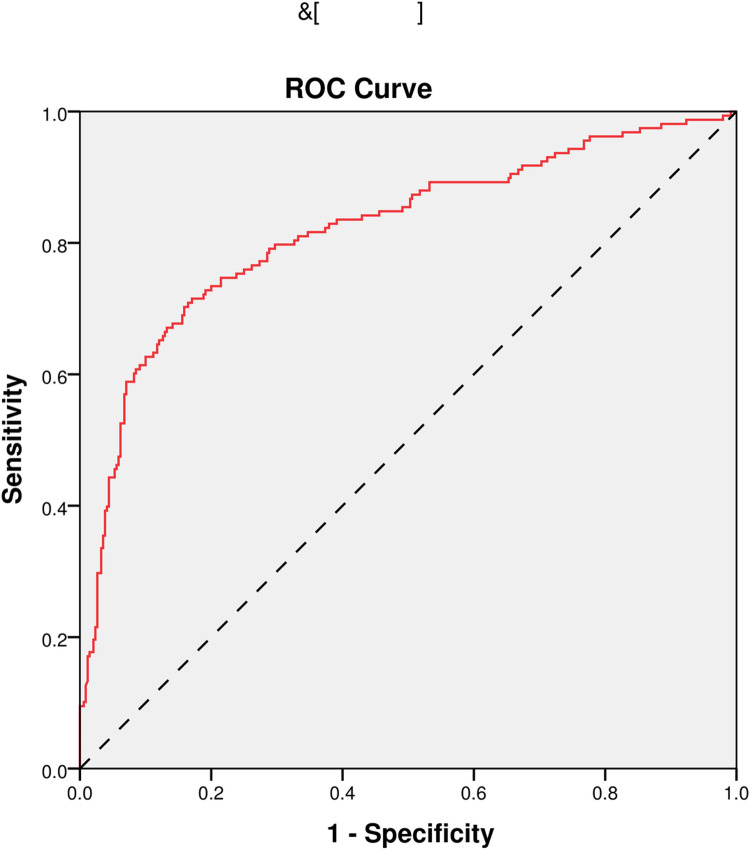
ROC curve of the model. ROC, receiver-operating characteristic.

In the end, we performed ROC analyses on age, NIHSS, pulse, and the model. And, the results that different predictors and the model all could predict ICHAP (P < 0.001) were shown in Figs 2–5. The area under the curve of the model (0.819) was higher than that of age (0.670), NIHSS (0.761), and pulse (0.609) (Table 4). Therefore, the model showed relatively higher prediction accuracy.

**Table 4:** Predictive effects of different factors on ICHAP.

	AUC	95%CI	P Value
Age	0.670	0.617-0.723	<0.001
NIHSS	0.761	0.714-0.808	<0.001
Pulse	0.609	0.555-0.663	<0.001
Model	0.819	0.776-0.862	<0.001

*NIHSS, National Institutes of Health Stroke Scale; AUC, area under the curve; CI, confidence interval.*

## Discussion

In this study, we found that a higher pulse was associated with ICHAP. Further, a higher pulse at admission might be used to predict the occurrence of ICHAP. We speculated that there were two possible reasons. Firstly, tachycardia has been proven to be one of the markers of sepsis, and rapid pulse rate often indicated increased cardiac output and abnormal cardiac function caused by sepsis [21]. Therefore, we suspected that rapid a pulse rate was associated with infection. And, a previous study had shown that when the pulse rate was greater than 111, the risk of post-stroke pneumonia in patients would increase significantly [22]. In addition, a recent study found that a faster heart rate at admission was positively correlated with stroke-associated pneumonia [23]. The results of these studies were similar to ours. Secondly, susceptibility to infections after stroke was associated with immunosuppression induced by central nervous system damage. When the central nervous system is damaged by stroke, the systemic cellular immune response is downregulated [24–26]. And, the cytokines released by the immune system can be regulated by humoral and autonomic nervous pathways [26,27]. Therefore, the dysfunction of the autonomic nervous system was partly responsible for immunosuppression. And, a study showed that autonomic dysfunction after intracerebral hemorrhage was associated with post-stroke immunosuppression and infection [28]. Moreover, a higher pulse which was one of the clinical markers of vagus nerve and sympathetic nerve function disorder was related to potential infection, systemic inflammatory response syndrome, and the risk of severe sepsis [23]. Therefore, we believe that pulse was a noninvasive diagnostic tool to detect stroke-induced alterations in autonomic function and its impact on the development of infections. So, an increased pulse rate at admission might be a predictor of ICHAP.

It has been confirmed by many studies that age is a risk marker for ICHAP because elderly patients are more likely to have more complications and more severe stroke which could cause a higher risk of infections [29]. And, they generally have decreased physical function and poor resistance to infections. This is also a reason why elderly patients are prone to ICHAP [30]. This was consistent with our results.

NIHSS and dysphagia were also independent predictors of ICHAP, similar to many previous studies. Higher NIHSS scores often implied poorer initial functional status, a higher risk of bedridden patients, and a greater need for ongoing post-stroke care. And, it also indicated that brain tissue damage caused by intracerebral hemorrhage was more serious, thus causing more severe immunosuppression and more prone to infections. So in these cases, elevated NIHSS could be used to predict ICHAP [6,31,32]. Dysphagia was a common complication in patients with intracerebral hemorrhage, especially in the brainstem or with a large amount of bleeding. Studies have shown that the risk of pneumonia in patients with dysphagia was three times higher than that in patients without dysphagia [33]. On the one hand, dysphagia was prone to aspiration; on the other hand, dysphagia often led to insufficient nutrient intake and increased risk of malnutrition. All of these situations could increase the risk of pneumonia [34].

In addition, we designed a model that might be used to predict ICHAP. The model included age, NIHSS, dysphagia, and pulse. Among them, age, NIHSS, dysphagia have been proven by many studies to be used in the prediction of SAP [6,29,33]. These results were repeated in our trial. To our knowledge, pulse might be used to participate in the ICHAP prediction for the first time. The possible reasons for its involvement are explained in the above paragraphs.

There were some shortcomings in our study. Firstly, our sample size was not large enough, which might lead to potential selectivity bias. Secondly, admission time may lead to bias. All patients in this study were admitted within 48 hours of onset, including some patients admitted 24 hours after the onset of stroke. However, every patient who had pre-stroke infections was ruled out on admission based on the patient’s symptoms and examination results in the emergency department. Therefore, the bias was within acceptable ranges. Thirdly, the blood test items included in our study did not contain some known indicators that were associated with ICHAP. There might be bias. Finally, our research was a single-center study, which might cause the sample to not fully reflect the overall situation.

## Conclusions

As far as we know, this study was the first to discover that a faster admission pulse might predict ICHAP. Moreover, we designed a practical and simple ICHAP prediction model which included age, NIHSS, dysphagia, and pulse. And, the model was detected to have good prediction accuracy. These findings might help early identification of high-risk patients who might develop pneumonia. And, it might provide a reference for the timely preventive use of antibiotics.

Further large-scale studies which should include a larger sample and combine all the reliable predictors are needed to confirm the relationship between pulse and ICHAP.

## Supporting information

S1 Data The research support data without unrecognizable information. (XLSX)

## Abbreviations

SAP: stroke-associated pneumonia
ICHAP: intracerebral hemorrhage-associated pneumonia
P: probability
ROC: receiver-operating characteristic
AUC: area under the curve
NIHSS: National Institute of Health Stroke Scale
mRS: modified Rankin Scale
CRP: C-reactive protein
MMSE: Mini-mental State Examination
MoCA: Montreal Cognitive Assessment
MRI: brain nuclear magnetic resonance
GCS: Glasgow Coma scale
WST: water swallow test
INR: International normalized ratio
SPSS: statistical product and service solutions
OR: odds ratio
CI: confidence interval
LDL-C: low-density lipoprotein cholesterol
TLR: cytokines and toll-like receptors
COPD: chronic obstructive pulmonary disease
B: regression coefficient
